# Target and Suspect Screening Reveal PFAS Exceeding European Union Guideline in Various Water Sources South of Lyon, France

**DOI:** 10.1021/acs.estlett.4c01126

**Published:** 2025-02-07

**Authors:** Termeh Teymoorian, Louis Delon, Gabriel Munoz, Sébastien Sauvé

**Affiliations:** † Département de chimie, Université de Montréal, Montréal, QC H2V 0B3, Canada; ‡ Ozon l’Eau Saine, Lyon, 69000, France; § Centre d’expertise en analyse environnementale du Québec, ministère de l’Environnement, de la Lutte contre les changements climatiques, de la Faune et des Parcs, Québec, QC G1P 3W8, Canada

**Keywords:** Fluoropolymer manufacture, PFAS, Precursors, France, Lyon, Drinking water framework directive

## Abstract

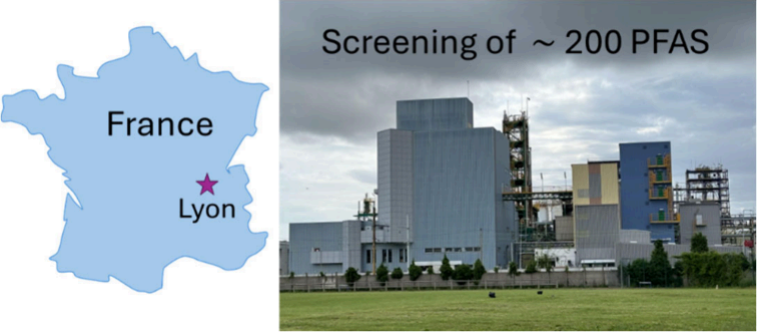

Lyon, a major hub for chemical industries in France, has been identified as a contamination hotspot of per- and polyfluoroalkyl substances (PFAS). Major chemical companies in the Pierre-Bénite area have used PFAS in the production of fluoropolymers and fluorotelomers, with effluents discharged into the Rhône River. This together with other contamination sources, such as firefighting foam use at a vicinal harbor oil depot, likely resulted in a complex PFAS signature. This study investigated PFAS contamination in various water sources in southern Lyon, including ponds, rivers, factory channels, wells, springs, and tap water. Out of 47 samples, 22 had a Σ_77_PFAS above 100 ng/L (maximum: ∼700 ng/L), and 67% of the tap water samples exceeded the European guideline of 100 ng/L for Σ_20_PFAS. Target PFAS profiles were dominated by perfluoroalkyl carboxylates (particularly C4 to C8), in agreement with their historical or current industrial usage. Suspect screening also revealed the occurrence of electrochemical fluorination precursors such as N-sulfopropyldimethylammoniopropyl perfluorohexanesulfonamido acetic acid (N-SPAmP-FHxSAA) and bistriflimide (used in the composition of ionic liquids). Certain fluorotelomers, including ESI+ (e.g., 6:2 fluorotelomer sulfonamidopropyl betaine (6:2 FTAB)) and ESI- (e.g., 6:2 FTS, 6:2 FTSAS-sulfone) compounds, were more prevalent in surface water than in tap or groundwater.

## 1 Introduction

Per- and polyfluoroalkyl substances (PFAS) are a vast group of chemicals used in industrial and consumer products.^1^ Long-chain perfluoroalkyl acids, including perfluorooctanoic acid (PFOA) and perfluorooctanesulfonate (PFOS), have been historically used in the production processes of fluoropolymers, in aqueous film-forming foams (AFFFs), and other use categories. Due to environmental and health concerns, these legacy PFAS are being replaced.^2^

PFAS pollution hotspots have resulted in the contamination of soil and water ecosystems^3^ and sometimes in harmful health effects in humans.^4^ Examples of major PFOA hot spots include one site along the Ohio River (West Virginia, United States)^5^ and sites in the Xiaoqing River basin (China),^6^ related to fluoropolymer manufacturing. Commercial airports and military bases are important PFOS contributors due to AFFF usage.^7−10^ A map of “forever pollution” in Europe was recently released, locating over 22,900 known and 21,400 presumptive contamination sites.^11^ According to the study, the European Union (EU) hosted at least 20 PFAS manufacturing facilities, among which 85% are still active and 25% are in France. Notably, two fluoropolymer production plants located in Pierre-Bénite (south of Lyon, France) are suspected of contributing to PFAS contamination.^12^

In 2012, a monitoring study conducted at French nationwide scale found unusually high levels of perfluoroalkyl carboxylic acids (PFCAs) in surface water downstream from Lyon.^13^ Miège et al. analyzed 14 PFAS in fish from the Rhône River near Lyon; unlike most studies on aquatic biota, where PFOS tends to dominate, perfluoroundecanoic acid (PFUnA) exhibited the highest concentrations.^14^ Dauchy evaluated 28 PFAS in soil and dust near a fluoroelastomer and polyvinylidene fluoride (PVDF) manufacture in Lyon. Elevated concentrations of long-chain PFCAs were detected downwind from the facility.^15^ The Direction Régionale de l’Environnement, de l’Aménagement et du Logement (DREAL) has monitored ∼25 PFAS in environments surrounding the Pierre-Bénite industrial complex, including food, home-grown garden products, and monthly industrial water discharges since 2022.^16^ However, much of the broader PFAS spectrum remains unexplored in the area.

Historically, the manufacture of fluoropolymers at Pierre-Bénite began in 1960 with polytetrafluoroethylene (PTFE). Between 1960 and 1986, approximately 26 to 52 tonnes of PFOA were used in the production of PTFE as an emulsion polymerization agent.^15,17^ In 1971, the Pierre-Bénite site became a pilot production facility for PVDF (Foreflon) and by 1980, its production capacity had reached 1500 tons per year. However, in the late 1980s, Foreflon was replaced by another PVDF product.^18^ From 2003 to 2017, a commercial product known as Surflon, containing a mixture of PFCAs with PFNA as the major component, was used in the PVDF production process. This product was later replaced by Capstone, a 6:2 FTSA-based product,^16,19^ in a different PVDF production process. Another company located at the Pierre-Bénite site utilized PFOA as a polymerization emulsion agent from 2004 to 2008, which has since been replaced by PFHxA.^15,16^ Despite commitments from both active chemical sites to treat PFAS-contaminated discharges (like the activated carbon treatment system by Arkema),^16^ concerns persist. Another potential source of PFAS contamination in this area is the historical fire at the oil depot of Lyon’s Port (Edouard Herriot) in 1987, where approximately 200 m^3^ of AFFF were applied during this event.^20^ The analysis of groundwater at Edouard Herriot Port revealed that firefighting foams primarily contained 6:2 fluorotelomer sulfonamidopropyl betaine (6:2 FTAB);^21^ however, this compound is not typically included in routine monitoring of water quality.

This study aims to extend our knowledge of the PFAS signature in water sources in southern Lyon, France. We analyzed 77 target and ∼120 suspect-target PFAS in water samples collected there in 2023–2024. The findings will provide critical baseline data ahead of upcoming European Union regulations set forth in Directive 2020/2184, which mandates compliance with parametric values of 500 ng/L for PFAS total (sum of all PFAS) and 100 ng/L for the sum of 20 PFAS by January 12, 2026. We also tested whether the European Union’s proposed list of 20 PFAS may (or may not) adequately represent the spectrum of PFAS present in these water sources. Additionally, specific regulatory actions are forthcoming for one of the fluorochemical sites in Pierre-Bénite. A prefectural decree mandates that the facility cease all PFAS use by 31 December 2024 and is required to progressively reduce its discharge of 6:2 FTS into the Rhône River, with a reduction of 80% by September 2024.^16^ The present study thus provides critical baseline data ahead of complete target reduction.

## 2 Methods and Materials

Details on chemicals and materials, including native PFAS and isotope-labeled standards, are provided in the Supporting Information (Text S1 and Tables S1–S2).

### Sample Collection

Lyon is France’s third-largest city and is situated at the confluence of the rivers Rhône and Saône. The Rhône Valley area is a key center for France’s chemical, pharmaceutical, and biotech industries. Water samples (n = 47) were collected in 2023–2024 from various sources, including ponds, channels, rivers, wells, spring water, and tap water, in southern Lyon (France), using precleaned 500 mL HDPE bottles.

### Chemical Analyses

Surface water and groundwater samples were passed through glass fiber filters (GFF; Advantec GF-75, 47 mm) prior to analysis, while tap water samples were unfiltered. Sample preparation involved spiking of water samples with surrogate internal standards prior to solid-phase extraction^22,23^ and analysis of 77 target PFAS by ultrahigh-performance liquid chromatography high-resolution mass spectrometry (UHPLC-HRMS Q-Exactive Orbitrap, Text S2 and Tables S3–S4). Suspect screening was conducted for ∼120 additional PFAS in negative and positive ionization modes (see also **Texts S3–S5**, Figures S1–S8, and Tables S5–S8). Suspect peaks were extracted within ±10 ppm of their theoretical *m*/*z*. Peaks with signal intensities below 1E4 were excluded, and any detections in blanks were subtracted from the samples using the average blank(s) from the corresponding batch results. We also used retention time patterns (i.e., increasing retention times with increasing fluoroalkyl chain length within each PFAS class), chromatographic peak shapes (i.e., presence of linear and branched isomers for ECF-based PFAS), and HRMS/MS spectral data. AFFF concentrates from different brands, including ECF-based (3 M LightWater) and fluorotelomer-based (Solberg Arctic Foam, Ansul Ansulite) formulations previously characterized by nontarget screening,^24^ were also enlisted to increase identification confidence. Suspects were assigned confidence levels based on the Schymanski scale.^25^

### QA/QC

Limits of detection (LODs) ranged between 0.002 and 0.57 ng/L (Text S6). Blanks (including method and trip blanks) were prepared using Milli-Q water and analyzed alongside field samples in each batch. Filter selection was based upon an earlier evaluation of over 50 PFAS in river matrix, with a mean filtration recovery of 87% (min-max = 64–104%; Table S9). Extracted calibration curves (mCAL) were generated using quite PFAS-free commercial bottled water, spiked with native PFAS and surrogates before SPE. These mCAL yielded determination coefficients (R^2^) consistently better than 0.9900, and were used for the quantification of QA/QC and field samples. The accuracy of the whole method (spikes before SPE) fell within the range of 70–130% in mineral bottled water and surface water (Text S6, Table S4, and Figure S9).^26,27^ QC samples were reanalyzed after every tenth water sample for continued calibration verification.

## 3 Results and Discussion

### 3.1 Target Screening

**Figure 1:**
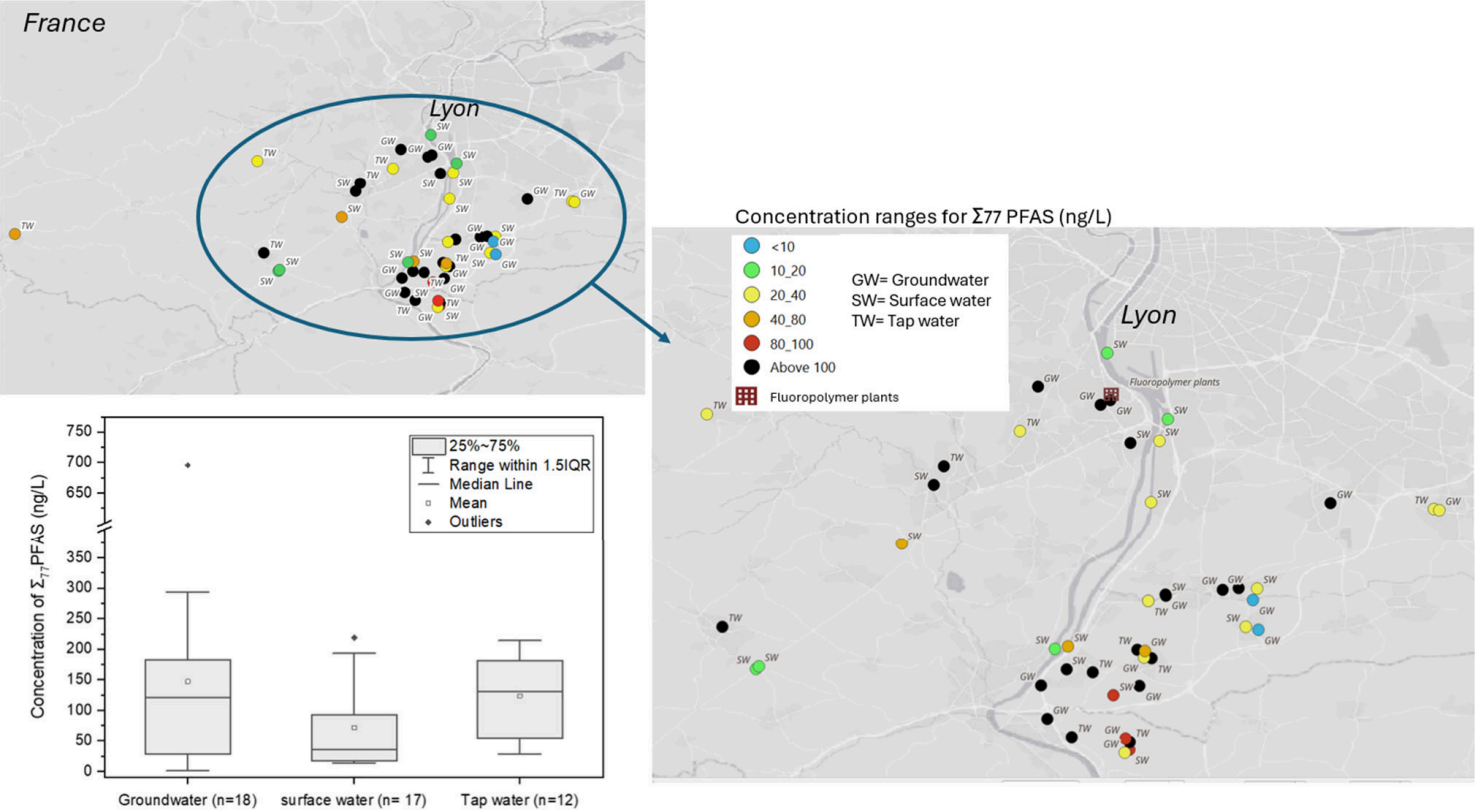
Spatial distribution of Σ_77_PFAS concentration ranges in water samples collected in southern Lyon, categorized in groundwater, surface water, and tap water with a corresponding box plot illustrating a statistical summary of total PFAS levels in each water source. On average, groundwater samples exhibited the highest Σ_77_PFAS concentrations (147 ng/L), followed by tap water (123 ng/L) and surface water (71 ng/L).

All samples were positive for the presence of target PFAS (Figure 1; see also Table S6 for summary statistics). Twenty-two out of 47 samples had total PFAS concentrations above 100 ng/L, primarily from groundwater and tap water samples. C4 to C9 PFCAs and C3 to C6 PFSAs had detection frequencies of 95% or higher. Among these, PFHxA (mean = 29.8 ng/L; median = 16.7 ng/L), PFPeA (19.5 ng/L; 14.2 ng/L) and PFOA (13.5 ng/L; 9.5 ng/L) typically exhibited the highest concentrations.

The average Σ_77_ PFAS was 147, 123, and 71 ng/L in groundwater, tap water, and surface water samples, respectively. Groundwater samples (n = 18), including wells and spring waters, had total PFAS concentrations ranging from 0.8 ng/L to 695 ng/L. The lowest PFAS concentration was from a well under 2 m of porous material, while the streamwater sample (L’Inverse) collected near this well had a total PFAS of 22.6 ng/L. This suggests that natural filtration may be effective in reducing PFAS concentrations. Two samples collected from wells (Well 1 & Well 2) located approximately 500 and 200 m from the fluoropolymer plant, had total PFAS concentrations of 266 ng/L and 149 ng/L, respectively. PFHxA was detected at concentrations of 87 ng/L and 35 ng/L, while PFOA was found at 56 ng/L and 21 ng/L in Well 1 and Well 2, respectively. The water from these wells was initially used for a vegetable garden but is now no longer used due to contamination by the nearby fluoropolymer manufacture. The highest level of Σ_77_ PFAS (695 ng/L) among all samples was recorded from a well located approximately 14 km from the fluoropolymer plant (Well 13). The supply for this well is drawn directly from the Rhône alluvial aquifer. The contamination observed is attributed to the diffusion of pollutants from the alluvial aquifer in the Pierre-Bénite area following decadal use and release.

**Figure 2:**
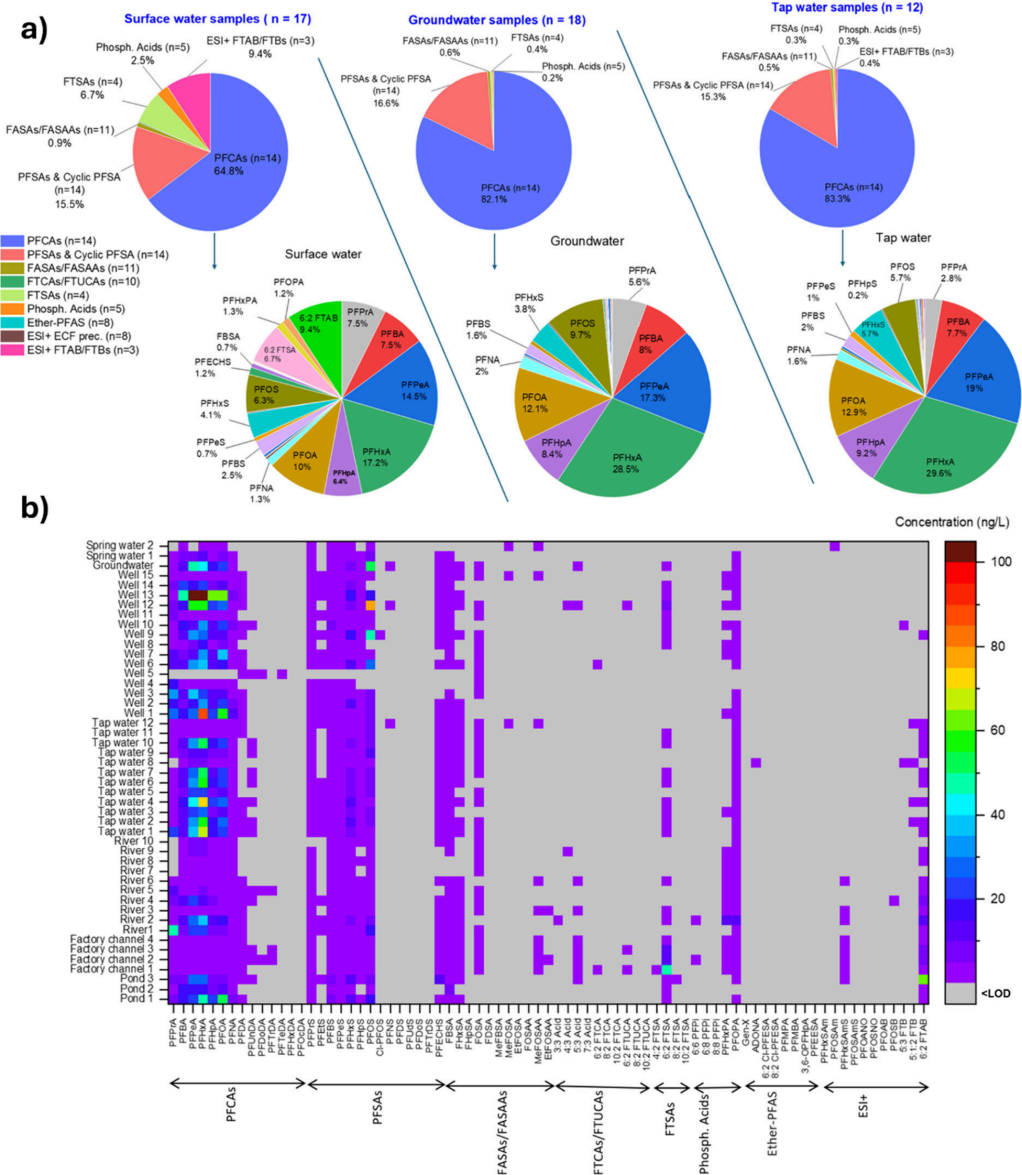
PFAS profiles in the collected water samples from southern Lyon. (a) Contribution percentage of different PFAS classes, into surface water, groundwater, and tap water. (b) Heatmap to show the concentration levels (ng/L) of all targeted PFAS across individual water samples from southern Lyon. The overall PFAS profile varied depending on the type of water but PFCAs followed by PFSAs were in all cases the major contributors.

Figure 2 presents the overall profile of different PFAS superclasses when grouping water samples by type (**2a**), and the corresponding heatmap with individual PFAS × individual samples (**2b**). PFCAs (mainly C3–C8) were the major contributors across all samples, especially in groundwater (PFCAs = 82%) and tap water (PFCAs = 83%) samples compared to surface water (PFCAs = 65%). ESI+ fluorotelomers, fluorotelomer sulfonic acids (FTSAs), and phosphonic acids were more prevalent in surface water than in tap water or groundwater.

Zwitterionic 6:2 FTAB had a much higher contribution in PFAS profiles of surface water samples (9.4%) compared to other water sources, as did 6:2 FTS (6.7% contribution for surface water samples). This distribution indicates potential pollution sources, such as the alluvial aquifer beneath Port Edouard Herriot for 6:2 FTAB. Given that 6:2 FTAB can undergo transformation to 6:2 FTS, and 6:2 FTS can further degrade into PFHxA and other PFCAs (Figure S10), this highlights another potential source of contamination for PFHxA, in addition to the industrial site. The highest level of 6:2 FTAB (62 ng/L) was recorded from a small pond connected to La Mouche River, close to the wastewater and rainwater purification plant (∼700 m). This finding is consistent with the observations of Boiteux et al. (2017) elsewhere in France.^28^ In their study, they observed that PFAS patterns in wells differed significantly from those in a river that received effluents from a fluorochemical industry. Boiteux et al. also highlighted that conventional drinking water treatments may not efficiently remove PFAS, and in some cases, processes like ozonation can even increase the concentration of specific PFAS, including the breakdown products of 6:2 FTAB.^28^

The maximum concentrations of two perfluoroalkyl phosphonic acids, PFHxPA (12 ng/L) and PFOPA (11 ng/L), were found in a sample collected from the Garon River (River 2; Σ_77_PFAS = 174 ng/L), a tributary of the Rhône River. The sample was collected approximately 7 km from the fluorochemical facility site, but to the best of our knowledge, it is unlikely to be hydro-geologically connected to the facility. Other sources than the fluorochemical manufacture might be responsible for this peculiar profile. For instance, C6–C12 PFPAs have been used as wetting agents in consumer applications and as wetting/antifoaming agents in pesticide formulations.^29^

### 3.2 Suspect Screening

An additional ∼120 PFAS were evaluated using suspect screening, leading to the identification of 49 additional suspects (see also Tables S7–S8). Approximately 98% of the samples tested positive for at least one suspect-target PFAS. The highest total concentration of suspect PFAS (22 ng/L) was observed in the well water sample that also had the highest level of the Σ_77_ PFAS (695 ng/L) among all samples and whose water supply is drawn directly from the Rhône alluvial aquifer.

Bistriflimide (used in the composition of ionic liquids) was found in 81% of the samples from the present study. It is commonly employed in various applications, including the production of lithium-ion batteries, and few studies reported this compound from landfill leachate^30^ and e-waste recycling facilities.^31^ In recent years, significant research has been conducted on the development of fluorinated lithium salts, which are crucial components in the manufacture of electrolytes for lithium-ion batteries.^32,33^ A production center is situated within a major research hub in the Rhône-Alpes region. To the best of our knowledge, this is the first report on this compound in surface and groundwater samples of France. The detection of bistriflimide in our study may be associated with recent developments in lithium-ion battery technologies.^34^ Guelfo et al. evaluated the presence of bis(trifluoromethylsulfonyl)imide (bis-FMeSI) in environmental samples, including surface water near PFAS production facilities in the USA and Europe.^34^ Bis-FMeSI concentrations were in the overall range of ∼2–2400 ng/L in surface water samples from the Minneapolis-St. Paul region near 3M’s manufacturing facility, ∼ 1–82 ng/L in Antwerp, Belgium (3M), and ∼4–7 ng/L in Salindres, France (Rhodia-Solvay).^34^

Short-chain PFAS such as FPrSA, FPeSA, NMeFPrSAA, N-SPAmP-FPrSAA, N-SPAmP-FBSAA, and N-SPAmP-FHxSAA (with detection frequencies of 66%, 66%, 81%, 32%, 49%, and 77%, respectively) are generally associated with ECF-based AFFF formulations. FPrSA and FPeSA were also recently reported in AFFF-impacted fish samples.^35,36^ While the concentrations of additional PFAS identified through suspect screening were relatively low (Figure S11), this approach provided insights on less frequently monitored compounds. The highest semiquantified concentrations were observed for NMeFBSAA (10.7 ng/L) and NMeFPrSAA (7.9 ng/L), both from well 13, followed by N-TAmP-FHxSAP (4.2 ng/L) from pond 3, and 6:2 FTSAS-sulfone (3.9 ng/L) from river 2.

### 3.3 Comparison with European Union and USEPA Guidelines

The percentage of tap water samples with Σ_20_PFAS concentrations above and below the European Union’s threshold of 100 ng/L is shown in the Supporting Information, Figure S12.a. A significant 67% (8 out of 12) of the samples exceeded this limit.^37^

The EU 20-PFAS list represented a high coverage (mean ∼90%) of our ΣPFAS (Figure S12.b). The observed results somewhat invalidate the initial hypothesis that the EU list of 20 PFAS might not suffice to attain a good PFAS coverage (Figure S12). This is explained by the fact that some industrial sites have a long history of use of old PFAS, such as PFOA and homologues, while other types of contaminated sites, such as AFFF use sites, may use formulations where precursors (omitted from the EU list) make up the bulk of the PFAS. Notably, no tap water samples exceeded the broader EU guideline value of 500 ng/L for total PFAS.

None of the surface water samples exceeded the European Union maximum allowable concentration-environmental quality standards (MAC-EQS) inland surface water level of 36000 ng/L for PFOS. However, all 17 samples surpassed the annual average (AA-EQS) level of 0.65 ng/L for PFOS (PFOS concentration range: 0.70–19 ng/L).^38^

Figure S13 compares the levels of five PFAS (PFOA, PFNA, PFHxS, PFOS, and Gen-X) in tap water samples and the US-EPA final maximum contaminant enforceable levels (MCLs) of 4 ng/L for PFOS and PFOA, and 10 ng/L for the rest of the three.^39^ While the concentrations of Gen-X (all nondetected) and PFNA were below the enforceable limits, regulatory thresholds for PFOA and PFOS were exceeded in 75% and 83% of the tap water samples, respectively. These findings provide a valuable database for future efforts to mitigate the environmental and public health impacts of PFAS pollution in Lyon and similar urban-industrial areas.

## Supporting Information Available

Additional information on methodology, quality control, figures, and tables related to target and suspect screening such as full scan UHPLC-HRMS chromatograms and high-resolution MS/MS spectra for selected PFAS and comparisons with US-EPA and EU guidelines (PDF)
Details for suspect screening and full results (XLSX)
