# Supplementary material for: Target and Suspect Screening Reveal PFAS Exceeding European Union Guideline in Various Water Sources South of Lyon, France
Source: Environ Sci Technol Lett. 2025 Feb 7;12(3):327–33. doi: 10.1021/acs.estlett.4c01126 (PMC11905281; doi:10.1021/acs.estlett.4c01126)
Supplement: Supplementary file 1 — ez4c01126_si_001.pdf [file ez4c01126_si_001.pdf]

Supporting Information

Target and Suspect Screening Reveal PFAS Exceeding European Union Guideline in Various Water Sources South of Lyon, France

Termeh Teymoorian[1], Louis Delon[2], Gabriel Munoz[3], Sébastien Sauvé[1]*

[1] Département de chimie, Université de Montréal, Montréal, QC, Canada.

[2] Ozon l'Eau Saine, Lyon, France.

[3] Centre d'expertise en analyse environnementale du Québec, ministère de l'Environnement, de la Lutte contre les changements climatiques, de la Faune et des Parcs, Québec, QC, Canada.

* Corresponding author: sebastien.sauve@umontreal.ca

Table of contents

SI Texts

Text S1. *Details on Chemicals and Materials*
Text S2. *Instrumental analysis*
Text S3. *Retention time confirmation for suspect PFAS using AFFF solutions*
Text S4. *Quantification confidence levels*
Text S5. *Summary of Schymanski confidence levels for substance identification*
Text S6. *QA/QC*

SI Tables

Table S1. *List of native PFAS standards included in the targeted LC-MS method.*
Table S2. *List of surrogate internal standards (IS).*
Table S3. *UHPLC-HRMS acquisition method.*
Table S4. *Mean whole-method accuracy % of the method (n=6), based on mineral bottled water matrix samples spiked with native PFAS and surrogate internal standards prior to automated SPE, and quantified against an extracted calibration curve (same as for field samples).*
Table S5. *Information on the foams used for suspect screening confirmation in this study.*
Table S6. *LODs, detection frequencies, and concentration range (minimum-maximum [ng/L] of values above LOD), median (ng/L), and mean (ng/L) of 77 target PFAS across water samples from Lyon, France.*
Table S7 (see Excel). *Details on the 121 PFAS in suspect screening including class, ion formula, ionization mode, theoretical m/z, observed m/z, mass error (δ ppm), reference calibrant, retention time, identification and quantification confidence levels, and detection frequency (%).*
Table S8 (see Excel). *Details of each PFAS concentration (ng/L) as well as sampling dates and coordinates of the collected samples.*
Table S9. *Filtration recovery data (%) of over 50 model PFAS in surface water matrix.*

SI Figures

Figures S1-S5. *Full Scan UHPLC-HRMS chromatograms and corresponding high-resolution MS/MS spectrum of Bistriflimide, 6:2 FTSAS-sulfone, NMeFPeSAA, UPFSA-3, and N-SPAmP-FHxSAA.*
Figure S6-S7. *Full-scan LC-HRMS chromatograms comparing retention times of example suspect PFASs in ESI– and ESI+ in water samples with those in AFFF samples, enhancing identification confidence. Representative AFFF samples included ECF-based (3M Light Water, n=2) and fluorotelomer-based (Solberg Arctic Foam, n=1, Ansul Ansulite, n=1) AFFFs analyzed at a 5000-fold dilution factor.*
Figure S8. *Structures of bistriflimide and other key PFAS classes analyzed in this study.*
Figure S9. *Accuracy of matrix spikes to surface water matrix.*
Figure S10. *Potential degradation transformation pathway of 6:2 FTAB into 6:2 FTS followed by further degradation into Short-Chain PFCAs.*
Figure S11. *Concentrations of Σ_{77} target PFAS (green bars) and Σ_{121} suspect PFAS (orange bars) across various water sources in southern Lyon. The red dashed line indicates the 100 ng/L*

threshold by the European Union, although this European Union limit applies specifically to 20 PFAS in drinking water.

Figure S12. a) *The doughnut chart shows the percentage of tap water samples with Σ_{20}PFAS concentrations above EU threshold (100 ng/L for Σ20PFAS);* **b)** *The bar charts illustrate the coverage percentages of the Σ_{20}PFAS from EU guideline for drinking water in comparison to our Σ_{198} target+suspect screening PFAS among the analyzed tap water samples in this study.*

Figure S13. *Concentrations of five PFAS compounds (PFOA, PFNA, PFHxS, PFOS, and Gen-X) in tap water samples. The red dashed line represents the US-EPA final maximum contaminant enforceable level (MCL) of 4 ng/L for PFOS and PFOA, while the blue dashed line indicates the US-EPA final MCL of 10 ng/L for PFHxS, PFNA, and Gen-X.*

Text S1. Details on Chemicals and Materials

Methanol, water with 0.1% formic acid, and acetonitrile, all in HPLC grade, were obtained from Fisher Scientific (ON, Canada). Additionally, NH_4OH was acquired from Sigma-Aldrich (MO, USA). Strata-X-AW cartridges (200 mg/6 mL) by Phenomenex (Torrance, CA, USA) were also purchased for the solid phase extraction (SPE) method.

Text S2. Instrumental analysis

PFAS detection was performed using ultra-high-performance liquid chromatography coupled with high-resolution mass spectrometry (UHPLC-HRMS) equipped with an electrospray ionization source operating in both positive and negative ionization modes. Chromatographic separation was achieved using a Thermo Hypersil Gold C18 column (100 mm × 2.1 mm, 1.9 µm particle size) maintained at a temperature of 40 °C. The mobile phases consisted of (A) 0.1% formic acid in HPLC-grade water and (B) 0.1% formic acid in acetonitrile. The analysis utilized a Dionex Ultimate 3000 LC system connected to a Q-Exactive Orbitrap mass spectrometer, with a mass scan range of m/z 150–1000 in full scan acquisition mode. Additional details are available in Table S3.

Text S3. Retention time confirmation for suspect PFAS using AFFF solutions

To improve the identification confidence of suspect PFASs in water samples, retention time references were established using diluted aqueous film-forming foam (AFFF) concentrates including one AFFF concentrates from Ansul (Ansulite #Dorval), one from Solberg (Arctic Foam #072) and two from 3M (Light Water #030 and #053). AFFF samples were analyzed at a dilution factor of 5000 times using the same instrumental method as for the water samples (Table S5). The presence of various types of PFAS in these firefighting foam solutions was previously documented by Liu et al. (2024) using targeted analysis, suspect screening, and non-targeted analysis [1].

The retention times of suspects in the water samples were cross-referenced with those observed in the AFFF solutions, allowing confirmation where alignment was observed. It is noted that injections of AFFF samples occurred on a different day than the analysis of water samples, resulting in a slight but consistent retention time shift of approximately +6 to +10 seconds for PFAS in AFFF samples compared to water samples.

Figures S5–S6 illustrate examples of suspect retention times confirmed by these AFFF solutions.

Text S4. Quantification confidence levels

The detected PFAS can be classified into three quantification confidence levels (see also Table S7):

Quantitative (Qn): Analytes for which a true certified native standard (listed in Table S1) and a matching IS, or at least a closely related IS, were used if available.
Qualitative (Ql): Suspect analytes lacking true native standards but quantified using a structurally similar compound with an identical or similar terminal functional group.
Screen data quality (Sc): Suspect analytes, lacking both true native standards and similar reference standards, were tentatively quantified based on the calibration curve of a PFAS that had the same ionization mode.

For instance, perfluoropropane sulfonamide (FPrSA), quantified using the C4 homolog perfluorobutane sulfonamide (FBSA) as the reference calibrant for semi-quantification, was classified as a *Qualitative (Ql)* analyte; while 6:2 FTSAS-sulfone, quantified using 6:2 FTS as the reference calibrant for semi-quantification, was classified as a Screen data quality (Sc) analyte. See Table S7 for details on other suspect-target PFAS.

Text S5. Summary of Schymanski confidence levels for substance identification

The Schymanski classification system for substance identification encompasses five levels [2], ranging from confirmed structure (Level 1) to unknown mass of interest (Level 5). These levels provide a framework to describe the confidence in identifying chemical substances based on available data and methodologies.

Level 1: The ideal scenario, where the substance identification is confirmed using a reference standard. Requires matching MS, MS/MS spectrum, and retention time data.

Level 2, Probable Structure:

- ***2a, Library Match:*** An exact structure is proposed by matching to literature or spectral library data.
- ***2b, Diagnostic Evidence****:* No other structure fits the experimental data, but no standard or literature matches exist. Diagnostic fragments or experimental context provide support.

Level 3, Tentative Candidate(s): Represents uncertainty, with multiple possible structures but insufficient data to pinpoint one, the Exact structure remains speculative

Level 4: Unequivocal Molecular Formula: A molecular formula is determined based on spectral data, structural identification is not possible due to insufficient MS/MS data.

Level 5: Mass of Interest: The exact mass (m/z) is measured but lacks information for assigning even a molecular formula.

Text S6. QA/QC

Limit of detection (LOD): The LOD was calculated by multiplying the standard deviation of the blanks by the Student's t coefficient for n-1 degrees of freedom at the 95% confidence level (where *n* represents the number of replicate blanks). Alternatively, for compounds not

present in blanks, the peak intensity of low-contaminated samples or calibration curve standards was used to estimate the LOD [3].

Accuracy: Whole-method accuracy replicates were assessed by spiking native PFAS and surrogate internal standards into quite PFAS-free commercially bottled water before extraction. These spiked replicates went through the same preparation and analytical method as the field water samples. Accuracy was calculated by comparing measured concentrations to expected values (Table S4).

Table S1. List of native PFAS standards included in the targeted LC-MS method.

Acronym	Name	Supplier	Ion mode
PFPrA	Perfluoropropanoic acid	Wellington Labs	ESI(-)
PFBA	Perfluorobutanoic acid	Wellington Labs	ESI(-)
PFPeA	Perfluoropentanoic acid	Wellington Labs	ESI(-)
PFHxA	Perfluorohexanoic acid	Wellington Labs	ESI(-)
PFHpA	Perfluoroheptanoic acid	Wellington Labs	ESI(-)
PFOA	Perfluorooctanoic acid	Wellington Labs	ESI(-)
PFNA	Perfluorononanoic acid	Wellington Labs	ESI(-)
PFDA	Perfluorodecanoic acid	Wellington Labs	ESI(-)
PFUnA	Perfluoroundecanoic acid	Wellington Labs	ESI(-)
PFDoA	Perfluorododecanoic acid	Wellington Labs	ESI(-)
PFTrDA	Perfluorotridecanoic acid	Wellington Labs	ESI(-)
PFTeDA	Perfluorotetradecanoic acid	Wellington Labs	ESI(-)
PFHxDA	Perfluorohexadecanoic acid	Wellington Labs	ESI(-)
PFOcDA	Perfluorooctadecanoic acid	Wellington Labs	ESI(-)

PFEtS	Perfluoroethane sulfonate	Apollo Scientific	ESI(-)
PFPrS	Perfluoropropane sulfonate	Wellington Labs	ESI(-)
PFBS	Perfluorobutane sulfonate	Wellington Labs	ESI(-)
PFPeS	Perfluoropentane sulfonate	Wellington Labs	ESI(-)
PFHxS	Perfluorohexane sulfonate	Wellington Labs	ESI(-)
PFHpS	Perfluoroheptane sulfonate	Wellington Labs	ESI(-)
PFOS	Perfluorooctane sulfonate	Wellington Labs	ESI(-)
Cl-PFOS	Chloroperfluorooctane sulfonate	Wellington Labs	ESI(-)
PFNS	Perfluorononane sulfonate	Wellington Labs	ESI(-)
PFDS	Perfluorodecane sulfonate	Wellington Labs	ESI(-)
PFUdS	Perfluoroundecane sulfonate	Wellington Labs	ESI(-)
PFDoS	Perfluorododecane sulfonate	Wellington Labs	ESI(-)
PFTrDS	Perfluorotridecane sulfonate	Wellington Labs	ESI(-)
FBSA	Perfluorobutane sulfonamide	Wellington Labs	ESI(-)
FHxSA	Perfluorohexane sulfonamide	Wellington Labs	ESI(-)
FHpSA	Perfluoroheptane sulfonamide	Wellington Labs	ESI(-)
FOSA	Perfluorooctane sulfonamide	Wellington Labs	ESI(-)
FDSA	Perfluorodecane sulfonamide	Wellington Labs	ESI(-)
MeFBSA	N-methyl-perfluorobutane sulfonamide	Wellington Labs	ESI(-)
MeFOSA	N-methyl-perfluorooctane sulfonamide	Wellington Labs	ESI(-)
EtFOSA	N-ethyl-perfluorooctane sulfonamide	Wellington Labs	ESI(-)
FOSAA	Perfluorooctane sulfonamidoacetic acid	Wellington Labs	ESI(-)
MeFOSAA	N-methyl-perfluorooctane sulfonamidoacetic acid	Wellington Labs	ESI(-)

EtFOSAA	N-ethyl-perfluorooctane sulfonamidoacetic acid	Wellington Labs	ESI(-)
3:3 acid	3:3 fluorotelomer carboxylic acid	Synquest	ESI(-)
4:3 acid	4:3 fluorotelomer carboxylic acid	Synquest	ESI(-)
5:3 acid	5:3 fluorotelomer carboxylic acid	DuPont USA	ESI(-)
7:3 acid	7:3 fluorotelomer carboxylic acid	DuPont USA	ESI(-)
6:2 FTCA	6:2 fluorotelomer carboxylic acid	Wellington Labs	ESI(-)
8:2 FTCA	8:2 fluorotelomer carboxylic acid	Wellington Labs	ESI(-)
10:2 FTCA	10:2 fluorotelomer carboxylic acid	Wellington Labs	ESI(-)
6:2 FTUCA	6:2 fluorotelomer unsaturated carboxylic acid	Wellington Labs	ESI(-)
8:2 FTUCA	8:2 fluorotelomer unsaturated carboxylic acid	Wellington Labs	ESI(-)
10:2 FTUCA	10:2 fluorotelomer unsaturated carboxylic acid	Wellington Labs	ESI(-)
4:2 FTSA	4:2 fluorotelomer sulfonate	Wellington Labs	ESI(-)
6:2 FTSA	6:2 fluorotelomer sulfonate	Wellington Labs	ESI(-)
8:2 FTSA	8:2 fluorotelomer sulfonate	Wellington Labs	ESI(-)
10:2 FTSA	10:2 fluorotelomer sulfonate	Wellington Labs	ESI(-)
6:6 PFPi	Bis(perfluorohexyl)phosphinate	Wellington Labs	ESI(-)
6:8 PFPi	Perfluorohexylperfluorooctylphosphinate	Wellington Labs	ESI(-)
8:8 PFPi	Bis(perfluorooctyl)phosphinate	Wellington Labs	ESI(-)
6:2 Cl-PFESA	6:2 chlorinated perfluoroalkylether sulfonate	Wellington Labs	ESI(-)
8:2 Cl-PFESA	8:2 chlorinated perfluoroalkylether sulfonate	Wellington Labs	ESI(-)
Gen-X (HFPO-DA)	Hexafluoropropylene oxide dimer acid	Wellington Labs	ESI(-)
ADONA	Dodecafluoro-3H-4,8-dioxanonanoate	Wellington Labs	ESI(-)
PFMPA	Perfluoro-4-oxapentanoic acid	Wellington Labs	ESI(-)
PFMBA	Perfluoro-5-oxahexanoic acid	Wellington Labs	ESI(-)
3,6-OPFHpA	Perfluoro-3,6-dioxaheptanoic acid	Wellington Labs	ESI(-)

PFEESA	Perfluoro(2-ethoxyethane)sulfonate	Wellington Labs	ESI(-)
PFECHS	Perfluoro-4-ethylcyclohexane sulfonate	Wellington Labs	ESI(-)
PFHxPA	Perfluorohexylphosphonic acid	Wellington Labs	ESI(-)
PFOPA	Perfluorooctylphosphonic acid	Wellington Labs	ESI(-)
AmPr-FHxSA (PFHxSAm)	Perfluorohexane sulfonamidopropyl amine	Wellington Labs	ESI(+)
AmPr-FOSA (PFOSAm)	Perfluorooctane sulfonamidoalkyl amine	Fluobon	ESI(+)
T-AmPr-FHxSA (PFHxSAmS)	Perfluorohexane sulfonamidopropyl ammonium	Wellington Labs	ESI(+)
T-AmPr-FOSA (PFOSAmS)	Perfluorooctane sulfonamidopropyl ammonium	Fluobon	ESI(+)
CMeAmPr-FOAd (PFOAB)	Perfluorooctane amidopropyl betaine	Fluobon	ESI(+)
CMeAmPr-FOSA (PFOSB)	Perfluorooctane sulfonamidopropyl betaine	Fluobon	ESI(+)
OAmPr-FOAd (PFOANO)	Perfluorooctane amidopropyl amine oxide	Fluobon	ESI(+)
OAmPr-FOSA (PFOSNO)	Perfluorooctane sulfonamidopropyl amine oxide	Fluobon	ESI(+)
5:3 FTB	5:3 fluorotelomer betaine	Wellington Labs	ESI(+)
5:1:2 FTB	5:1:2 fluorotelomer betaine	Wellington Labs	ESI(+)
6:2 FTSA-PrB (6:2 FTAB)	6:2 fluorotelomer sulfonamidopropyl betaine	Wellington Labs	ESI(+)

Table S2. List of surrogate internal standards (IS).

**Acronym**	**Supplier**
$^{13}C_4$-PFBA	Wellington Labs
$^{13}C_5$-PFPeA	Wellington Labs
$^{13}C_5$-PFHxA	Wellington Labs
$^{13}C_4$-PFHpA	Wellington Labs
$^{13}C_8$-PFOA	Wellington Labs
$^{13}C_9$-PFNA	Wellington Labs
$^{13}C_6$-PFDA	Wellington Labs
$^{13}C_7$-PFUnA	Wellington Labs
$^{13}C_2$-PFDoA	Wellington Labs
$^{13}C_2$-PFTeDA	Wellington Labs
$^{13}C_3$-PFBS	Wellington Labs
$^{13}C_3$-PFHxS	Wellington Labs
$^{13}C_8$-PFOS	Wellington Labs
$^{13}C_8$-FOSA	Wellington Labs
d_3-N-MeFOSA	Wellington Labs
d_5-N-EtFOSA	Wellington Labs
d_3-N-MeFOSAA	Wellington Labs
d_5-N-EtFOSAA	Wellington Labs
$^{13}C_2$-6:2 FTCA	Wellington Labs
$^{13}C_2$-8:2 FTCA	Wellington Labs
$^{13}C_2$-10:2 FTCA	Wellington Labs
$^{13}C_2$-6:2 FTUCA	Wellington Labs
$^{13}C_2$-8:2 FTUCA	Wellington Labs

$^{13}C_2$-10:2 FTUCA	Wellington Labs
$^{13}C_2$-6:2 FTSA	Wellington Labs
$^{13}C_2$-8:2 FTSA	Wellington Labs
$^{13}C_3$-HFPO-DA	Wellington Labs
TAmPr-FOAd (PFOAAmS)	Beijing Surfactant Institute

Note: The study acknowledges the limitation of the limited availability of isotope-labeled ESI+ PFAS standards at the time of analysis. For this purpose, a custom-synthesized standard (PFOAAmS) was employed as a surrogate internal standard for ESI+ PFAS. The choice of PFOAAmS is supported by literature evidence that amide-based PFAS are less frequent than sulfonamide-based one at PFAS-impacted sites (Nickerson et al. 2020 [4]; Liu et al. 2021 [5]), and by the lack of detectable levels of PFOAAmS when screened as a target PFAS in France [6], reducing the risk of this internal standard already occurring in the field samples prior spiking it. We also spiked PFOAAmS at a high enough level in the samples so that an interference from the sample itself would not be expected. This approach was specifically chosen to correct ESI+ PFAS for which no suitable isotope-labeled standards were commercially available at the time. After the study was conducted, an ESI+ PFAS isotope-labelled standard became available (13C3-6:2 FTAB).

Table S3. UHPLC-HRMS acquisition method.

**Instrument**	Thermo Q-Exactive Orbitrap mass spectrometer Dionex Ultimate 3000 UHPLC chain
**Analytical column**	Thermo Hypersil Gold C18 column (100 mm × 2.1 mm; 1.9 µm particle size)
**Delay column**	Thermo Hypercarb column (20 mm × 2.1 mm; 7 µm particle size)
**Column Temperature**	40°C
**Ionization**	Heated electrospray ionization source, negative and positive ion modes
**Acquisition modes**	Full Scan MS<br>t-MS^2 mode (Normalized collision energy (NCE); tested at different levels: 15−40%.)
**Mobile Phases**	A: 0.1% formic acid in HPLC-water
	B: 0.1% formic acid in acetonitrile
**Flow rate**	0.55 mL/min
**Injection Volume**	20 µL
**Orbitrap parameters**	Resolution setting 70,000 at *m/z* 200
	Scan range (*m/z*) 150–1000

Table S4. Mean whole-method accuracy % of the method (n=6), based on mineral bottled water matrix samples spiked with native PFAS and surrogate internal standards prior to automated SPE, and quantified against an extracted calibration curve (same as for field samples).

	Mean accuracy %	RSD%
**PFPrA**	108.2	8.0
**PFBA**	96.4	2.9
**PFPeA**	96.5	6.0
**PFHxA**	95.4	7.0
**PFHpA**	96.4	5.6
**PFOA**	95.8	6.5
**PFNA**	94.8	5.1
**PFDA**	92.8	4.8
**PFUnDA**	93.3	4.6
**PFDoDA**	99.8	4.0
**PFTrDA**	99.9	7.0
**PFTeDA**	100.5	2.2
**PFHxDA**	103.8	2.4
**PFOcDA**	94.3	18.6
**PFPrS**	92.4	3.8
**PFEtS**	92.1	9.2
**PFBS**	93.8	4.4
**PFPeS**	96.5	4.8
**PFHxS**	94.0	4.8
**PFHpS**	83.8	15.0
**PFOS**	94.6	4.5
**PFNS**	98.6	4.5
**PFDS**	108.0	13.5
**PFUdS**	109.2	18.4
**PFDoS**	113.4	20.7
**PFTrDS**	109.6	25.9
**FBSA**	104.5	9.3
**FHxSA**	104.8	3.1
**FHpSA**	103.4	6.9
**FOSA**	95.8	4.3
**FDSA**	109.2	11.6
**MeFBSA**	105.7	17.6
**MeFOSA**	90.5	3.3
**EtFOSA**	91.8	3.8
**FOSAA**	86.1	13.8
**MeFOSAA**	94.8	6.9

**EtFOSAA**	92.7	7.2
**3:3 Acid**	99.3	4.8
**4:3 Acid**	96.0	6.8
**5:3 Acid**	106.4	16.3
**7:3 Acid**	92.8	13.8
**6:2 FTCA**	96.2	8.3
**8:2 FTCA**	97.5	5.6
**10:2 FTCA**	101.9	9.6
**6:2 FTUCA**	98.1	5.6
**8:2 FTUCA**	98.9	5.9
**10:2 FTUCA**	106.5	10.5
**4:2 FTSA**	97.9	11.4
**6:2 FTSA**	92.5	5.4
**8:2 FTSA**	90.6	5.6
**10:2 FTSA**	102.2	17.5
**6:6 PFPi**	94.3	19.2
**6:8 PFPi**	92.8	11.1
**8:8 PFPi**	92.0	12.8
**PFHxPA**	92.9	8.3
**PFOPA**	89.5	11.6
**PFECHS**	82.5	13.2
**Gen-X**	91.5	8.3
**ADONA**	94.0	4.1
**6:2 Cl-PFESA**	94.5	5.4
**8:2 Cl-PFESA**	109.7	15.6
**PFMPA**	94.5	7.2
**PFMBA**	93.9	4.6
**3,6-OPFHpA**	93.5	7.1
**PFEESA**	90.1	2.0
**PFHxSAm**	95.5	9.9
**PFOSAm**	100.9	10.5
**PFHxSAmS**	96.0	10.5
**PFOSAmS**	104.7	10.8
**PFOANO**	102.4	10.1
**PFOSNO**	98.8	10.0
**PFOAB**	94.5	6.9
**PFOSB**	93.4	11.3
**5:3 FTB**	94.3	11.2
**5:1:2 FTB**	95.9	11.5

**6:2 FTAB**	93.8	8.6
**Cl-PFOS**	99.7	4.6

Table S5. Information on the foams used for suspect screening confirmation in this study.

Foam type	Foam sample name	Manufacturer	Use
PFAS-containing FT-based AFFF	Ansulite #Dorval	Canadian supplier	For extinguishing
	ArcticFoam072	European Supplier	
PFAS-containing ECF-based AFFF	Lightwater Foam030		
	Lightwater Foam053		

Table S6. LODs, detection frequencies, and concentration range (minimum-maximum [ng/L] of values above LOD), median (ng/L), and mean (ng/L) of 77 target PFAS across water samples from Lyon, France. Note: levels below LODs were considered as zero for statistical calculations in this study.

Compound	LOD (ng/L)	Min-Max (ng/L)	Median (ng/L)	Mean (ng/L)	Detection Frequency (%)
**PFPrA**	0.18	0.34-46	2.68	5.99	85.1
**PFBA**	0.020	0.48-50	8.60	8.86	97.9
**PFPeA**	0.029	0.63-143	14.19	19.45	95.7
**PFHxA**	0.017	0.11-322	16.66	29.79	97.9
**PFHpA**	0.019	0.07-62	7.21	9.27	97.9
**PFOA**	0.004	0.071-61	9.52	13.49	97.9
**PFNA**	0.014	0.05-14	1.11	1.95	95.7
**PFDA**	0.020	0.034-1.8	0.08	0.20	63.8
**PFUnDA**	0.034	0.058-1.0	—	0.14	29.8
**PFDoDA**	0.032	0.097-0.3	—	0.01	6.4
**PFTrDA**	0.036	0.042-0.1	—	<LOD	6.4
**PFTeDA**	0.028	0.13	—	<LOD	2.1
**PFHxDA**	0.050	—	—	—	—
**PFOcDA**	0.57	—	—	—	—
**PFPrS**	0.012	0.028-1.9	0.32	0.40	95.7
**PFEtS**	0.036	0.043-0.38	—	0.07	46.8
**PFBS**	0.008	0.064-9.9	1.72	2.19	97.9
**PFPeS**	0.008	0.028-3.3	0.51	0.86	97.9
**PFHxS**	0.005	0.053-19	2.68	4.99	97.9
**PFHpS**	0.009	0.032-1.4	0.17	0.25	87.2
**PFOS**	0.011	0.052-76	3.64	8.85	91.5
**PFNS**	0.023	0.046-0.1	—	<LOD	6.4
**PFDS**	0.027	—	—	—	—
**PFUdS**	0.032	—	—	—	—
**PFDoS**	0.020	—	—	—	—
**PFTrDS**	0.027	—	—	—	—
**FBSA**	0.010	0.016-5.8	0.17	0.39	89.4
**FHxSA**	0.009	0.009-0.65	0.03	0.08	63.8
**FHpSA**	0.013	—	—	—	—
**FOSA**	0.025	0.026-1.9	0.05	0.13	70.2
**FDSA**	0.029	—	—	—	—
**MeFBSA**	0.012	—	—	—	—
**MeFOSA**	0.023	0.15-0.27	—	0.01	6.4
**EtFOSA**	0.026	—	—	—	—

**FOSAA**	0.030	—	—	—	—
**MeFOSAA**	0.006	0.13-0.99	—	0.08	19.1
**EtFOSAA**	0.050	0.096-0.28	—	0.01	4.3
**3:3 Acid**	0.098	0.60	—	0.01	2.1
**4:3 Acid**	0.079	0.12-0.13	—	0.01	4.3
**5:3 Acid**	0.042	0.048-0.73	—	0.05	19.1
**7:3 Acid**	0.065	—	—	—	—
**6:2 FTCA**	0.037	0.20-0.22	—	0.01	4.3
**8:2 FTCA**	0.027	—	—	—	—
**10:2 FTCA**	0.13	—	—	—	—
**6:2 FTUCA**	0.017	0.026-0.12	—	0.01	6.4
**8:2 FTUCA**	0.027	—	—	—	—
**10:2 FTUCA**	0.046	—	—	—	—
**4:2 FTSA**	0.008	0.09	—	<LOD	2.1
**6:2 FTSA**	0.010	0.019-46	0.03	2.04	55.3
**8:2 FTSA**	0.017	0.08	—	<LOD	2.1
**10:2 FTSA**	0.047	—	—	—	—
**6:6 PFPi**	0.027	0.038-0.05	—	<LOD	4.3
**6:8 PFPi**	0.017	—	—	—	—
**8:8 PFPi**	0.044	—	—	—	—
**PFHxPA**	0.015	0.017-12	—	0.37	42.6
**PFOPA**	0.017	0.02-11	0.15	0.52	89.4
**PFECHS**	0.008	0.013-13	0.11	0.53	78.7
**Gen-X**	0.45	—	—	—	—
**ADONA**	0.014	0.07	—	<LOD	2.1
**6:2 Cl-PFESA**	0.018	—	—	—	—
**8:2 Cl-PFESA**	0.039	—	—	—	—
**PFMPA**	0.030	—	—	—	—
**PFMBA**	0.030	—	—	—	—
**3,6-OPFHpA**	0.048	—	—	—	—
**PFEESA**	0.008	—	—	—	—
**PFHxSAm**	0.015	—	—	—	—
**PFOSAm**	0.01	0.01	—	<LOD	2.1
**PFHxSAmS**	0.012	0.014-0.11	—	0.01	17.0
**PFOSAmS**	0.050	—	—	—	—
**PFOANO**	0.035	—	—	—	—
**PFOSNO**	0.056	—	—	—	—
**PFOAB**	0.072	—	—	—	—
**PFOSB**	0.122	0.13	—	<LOD	2.1
**5:3 FTB**	0.012	0.1	—	<LOD	4.3
**5:1:2 FTB**	0.012	0.024-1.0	—	0.03	8.5

**6:2 FTAB**	0.028	0.16-62	0.18	2.54	53.2
**Cl-PFOS**	0.002	0.13	—	<LOD	2.1

Table S7 (see Excel file). Details on the 121 PFAS in suspect screening including class, ion formula, ionization mode, theoretical m/z, observed m/z, mass error (δ ppm), reference calibrant, retention time, identification and quantification confidence levels, and detection frequency (%).

Table S8 (see Excel file). Details of each PFAS concentration (ng/L) as well as sampling dates and coordinates of the collected samples.

Table S9. Filtration recovery data (%) of over 50 model PFAS in surface water matrix.

	Filtration recovery % in surface water matrix	
	Mean (n = 3)	STDEV (n=3)
PFBA	92	9
PFPeA	97	1
PFHxA	95	3
PFHpA	94	5
PFOA	92	3
PFNA	89	2
PFDA	84	2
PFUnA	82	4
PFDoA	77	1
PFTrDA	69	0
PFTeDA	64	4
PFHxDA	72	4
PFPrS	83	2
PFBS	94	0
PFPeS	90	3
PFHxS	94	2
PFHpS	91	3
PFOS	88	3
PFNS	82	1
PFDS	80	5
PFDoS	74	3
PFECHS	92	3
FBSA	97	4
FHxSA	95	4
FOSA	84	5
MeFOSA	66	5
EtFOSA	67	2
FOSAA	93	9
EtFOSAA	78	2
3:3 acid	93	11
5:3 acid	99	8
7:3 acid	100	10
4:2 FTSA	101	4
6:2 FTSA	95	2
8:2 FTSA	84	1
10:2 FTSA	76	5
ADONA	92	9
6:2 Cl-PFESA	89	3
8:2 Cl-PFESA	82	6
Gen-X	94	4
Cl-PFOS	88	0
N-AP-FHxSA	99	20
N-TAmP-FHxSA	100	12
6:2 FTAB	93	9
5:3 FTB	90	5
5:1:2 FTB	86	6
PFOAB	93	15
PFOSB	65	9
PFOANO	104	15
PFOSNO	86	8
PFOSAm	65	17

PFOSAmS	70	10

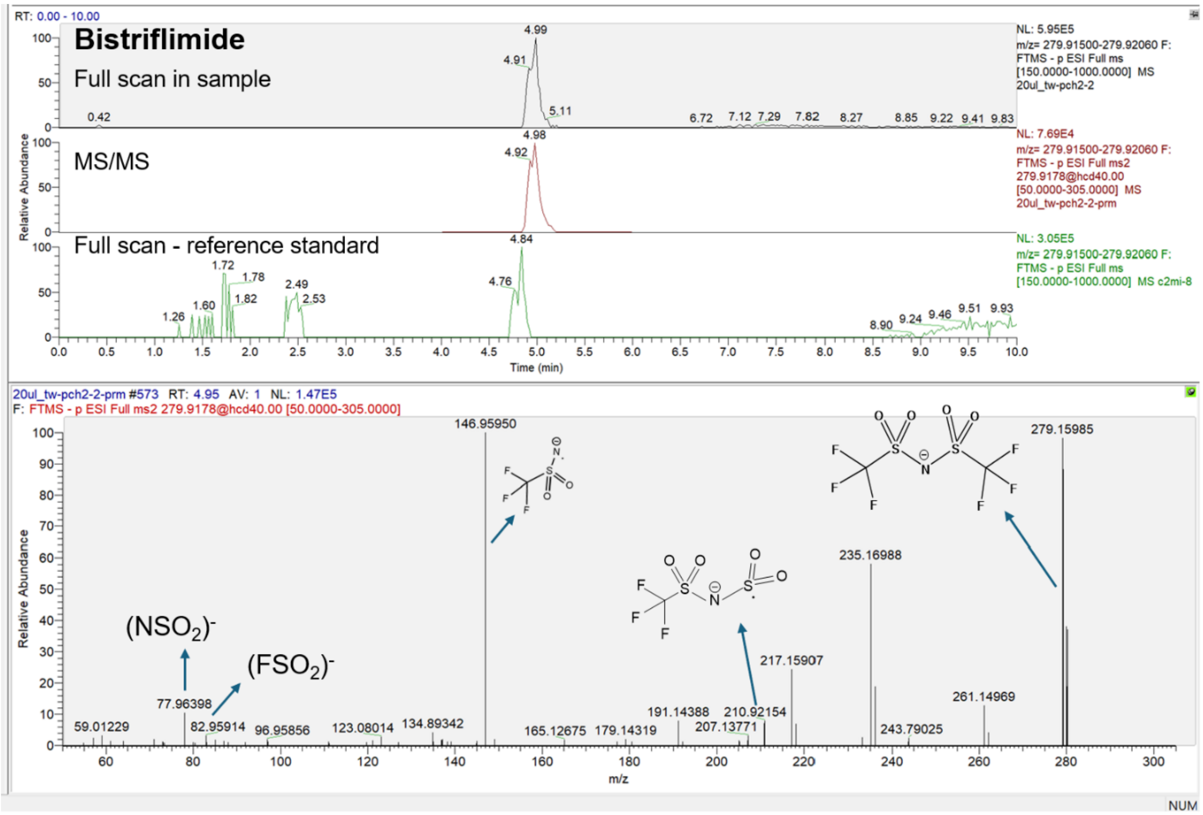

Figure S1. Full Scan UHPLC-HRMS chromatograms and corresponding high-resolution MS/MS spectrum of Bistriflimide (with a normalized collision energy of 40%) in the well 13 (PCH2-2) from Lyon, France, and the reference standard (injected on a different day). MS/MS fragment ions are also in agreement with the detected fragments by Barola et al. 2023 [7].

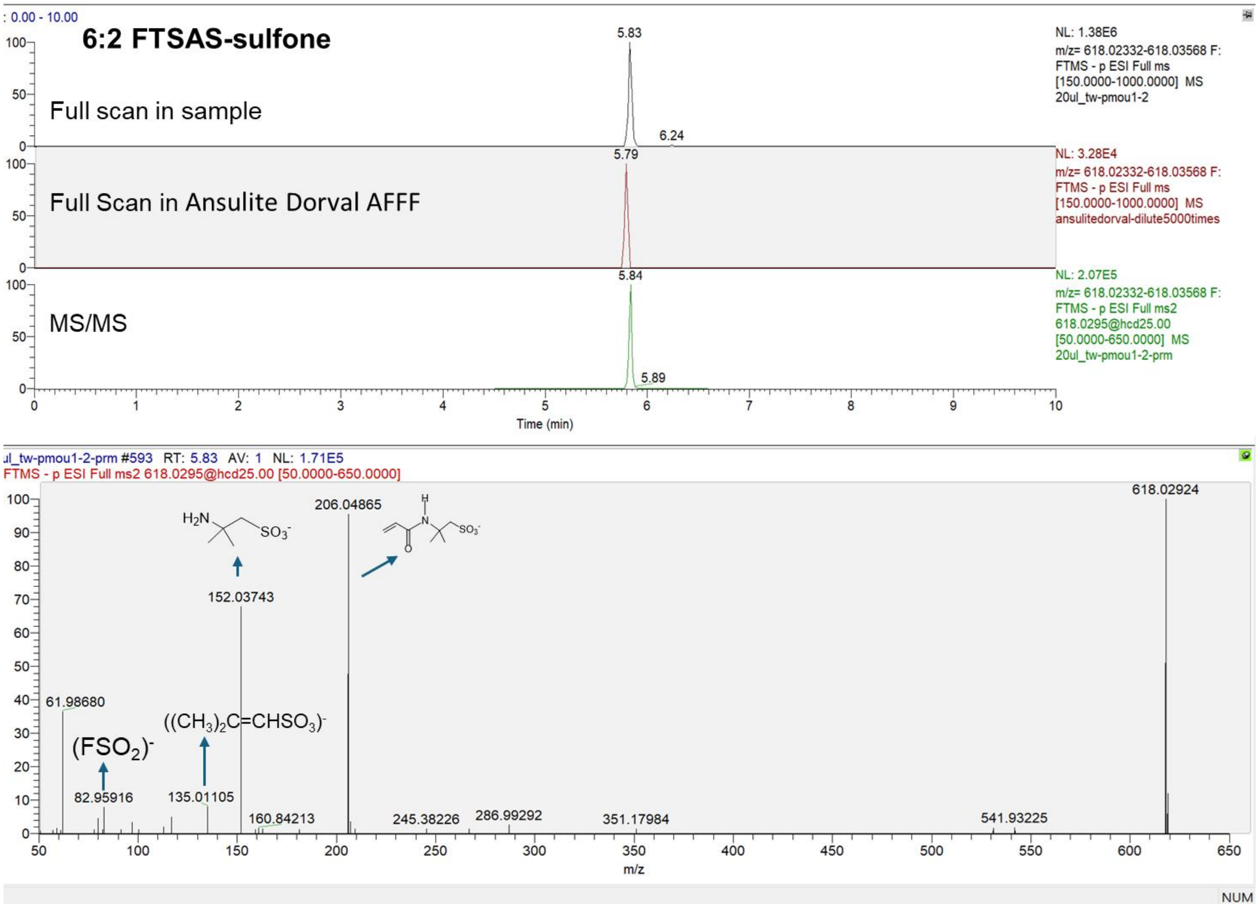

Figure S2. Full-scan UHPLC-HRMS chromatograms of a surface water sample (PMOU1-2) from Lyon, France, and Ansulite Dorval AFFF (diluted 5000 times). The corresponding high-resolution MS/MS spectrum of 6:2 FTSAS-sulfone, obtained at a normalized collision energy of 25%, is also shown. *Note:* The Ansulite Dorval AFFF sample was injected on a different day.

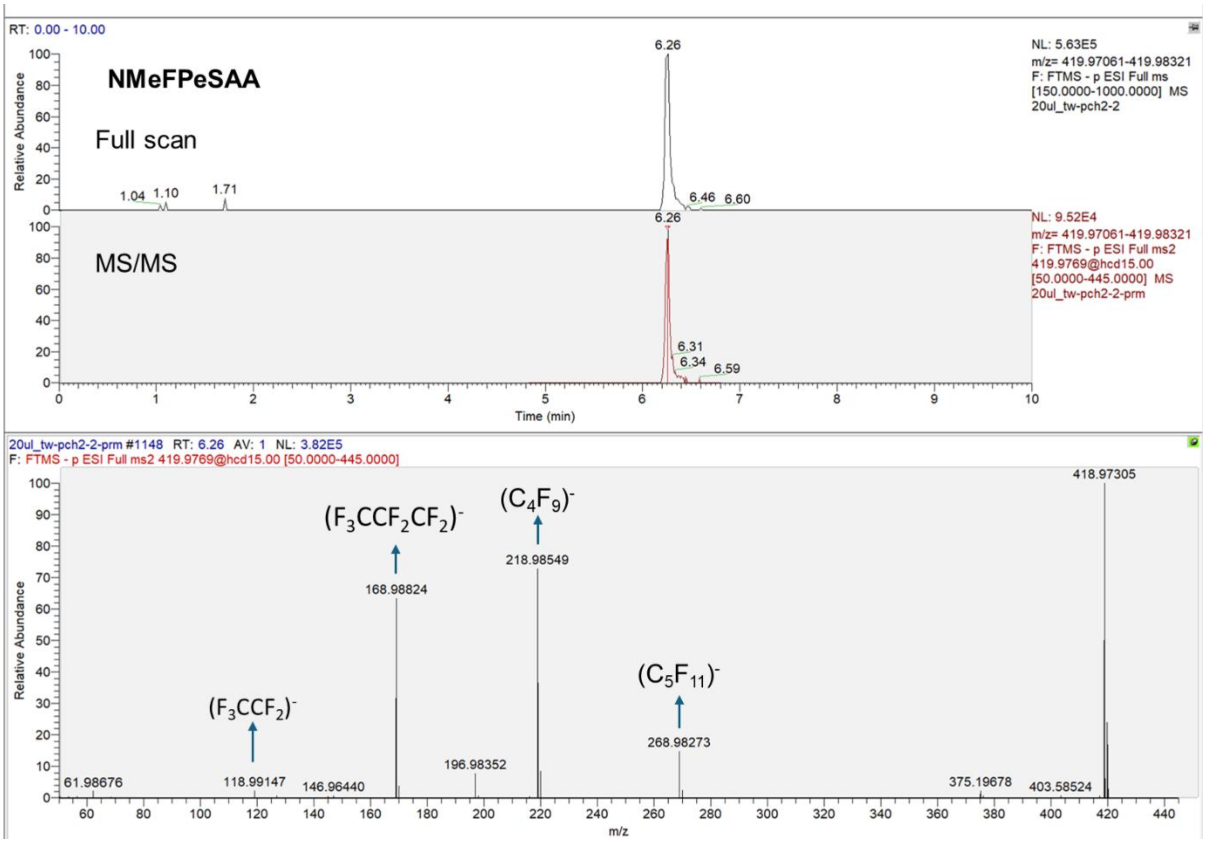

Figure S3. Full Scan UHPLC-HRMS chromatograms and corresponding high-resolution MS/MS spectrum of NMeFPeSAA with a normalized collision energy of 15% in the well sample 13 (PCH2-2) from Lyon, France.

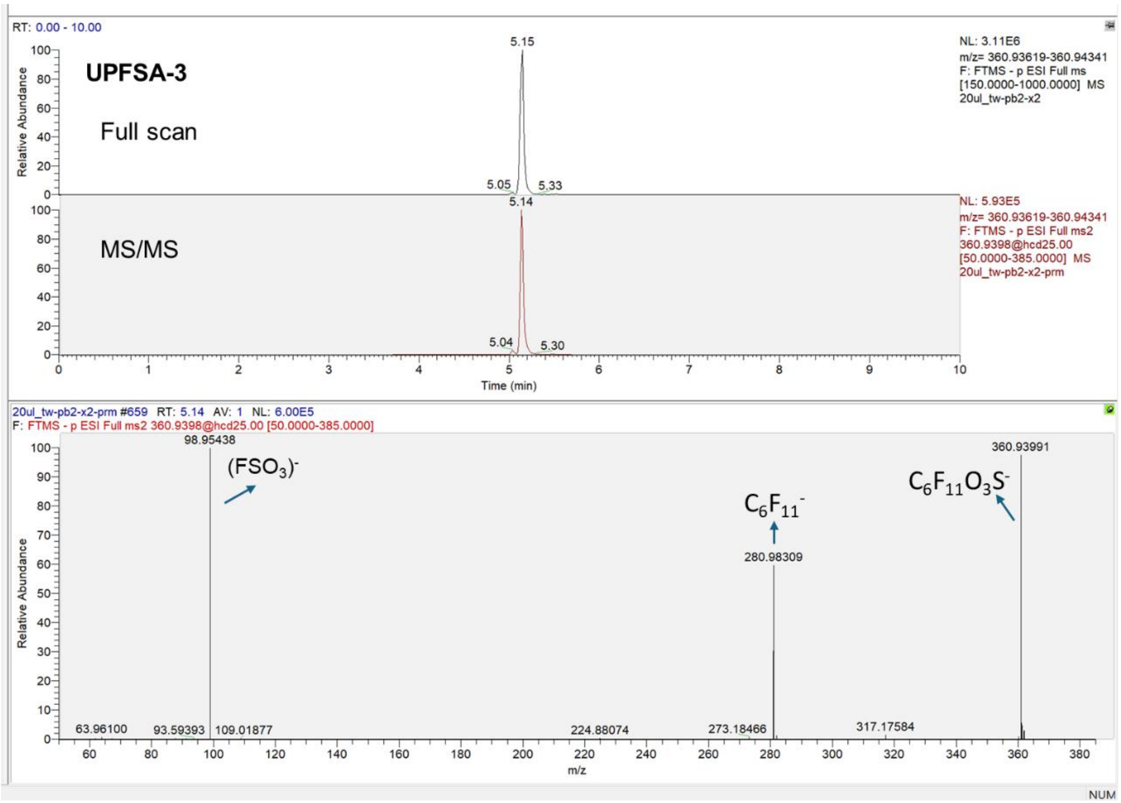

Figure S4. Full Scan UHPLC-HRMS chromatograms and corresponding high-resolution MS/MS spectrum of UPFSA-3 with a normalized collision energy of 25% in a well water sample 2 (PB2) from Lyon, France. MS/MS fragment ions are in agreement with a previous study by Chow et al. 2022 (unsaturated Perfluorohexane Sulfonate) [8].

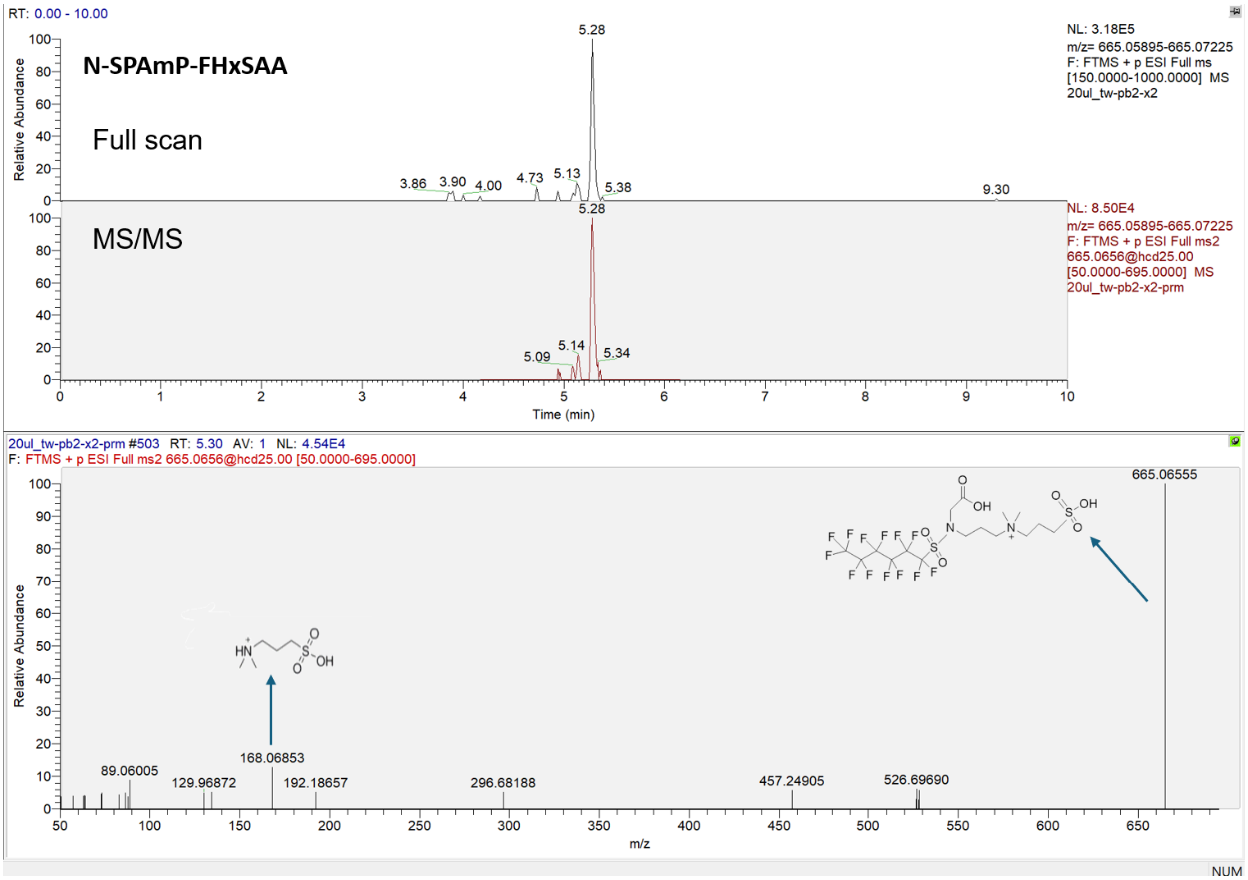

Figure S5. Full Scan UHPLC-HRMS chromatograms and corresponding high-resolution MS/MS spectrum of N-SPAmP-FHxSAA with a normalized collision energy of 25% in a well water sample 2 (PB2) from Lyon, France.

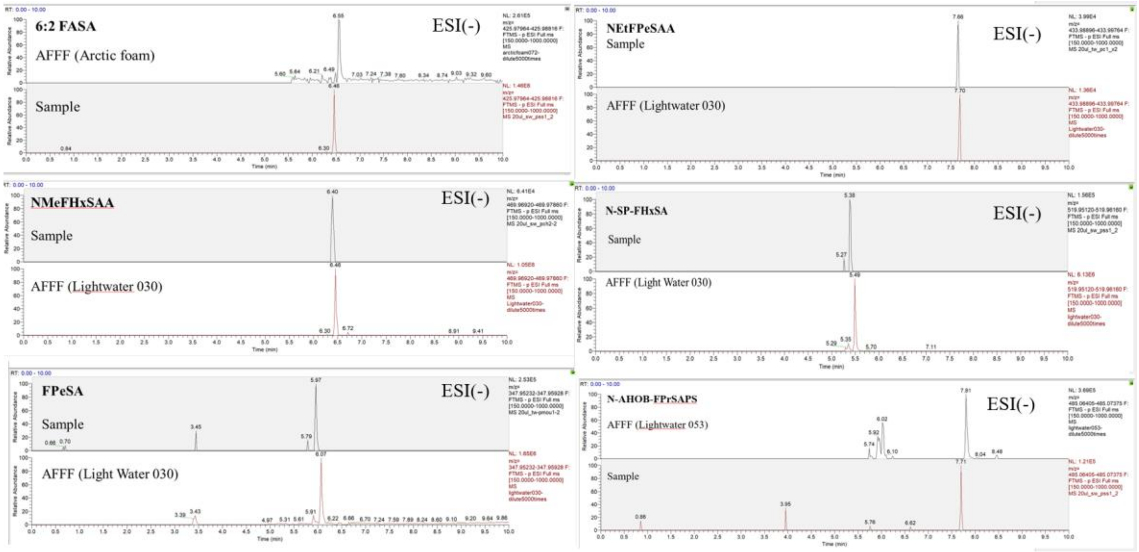

Figure S6. Full-scan LC-HRMS chromatograms comparing retention times of example suspect PFASs in ESI– in water samples with those in AFFF samples, enhancing identification confidence. Representative AFFF samples included ECF-based (3M Light Water) and fluorotelomer-based (Solberg Arctic Foam) analyzed at a 5000-fold dilution factor. The injections for AFFF and water samples were conducted on different days, resulting in a consistent retention time shift of approximately 6-10 seconds for AFFF samples.

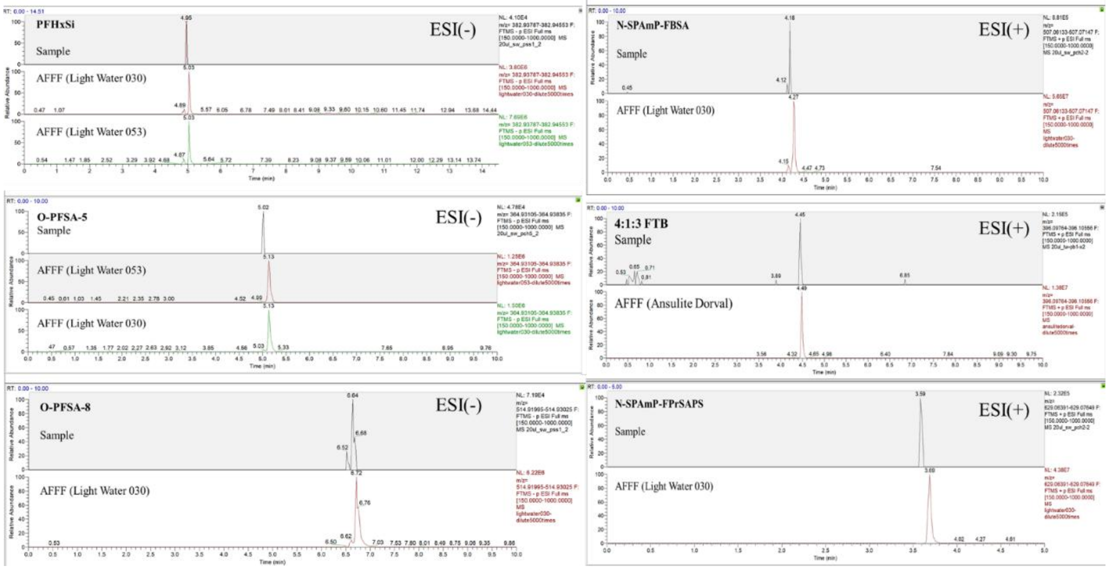

Figure S7. Full-scan LC-HRMS chromatograms comparing retention times of example suspect PFASs in ESI– and ESI+ in water samples with those in AFFF samples, enhancing identification confidence. Representative AFFF samples included ECF-based (3M Light Water, n=2) and fluorotelomer-based (Ansul Ansulite) AFFFs analyzed at a 5000-fold dilution factor. The injections for AFFF and water samples were conducted on different days, resulting in a consistent retention time shift of approximately 6-10 seconds for AFFF samples.

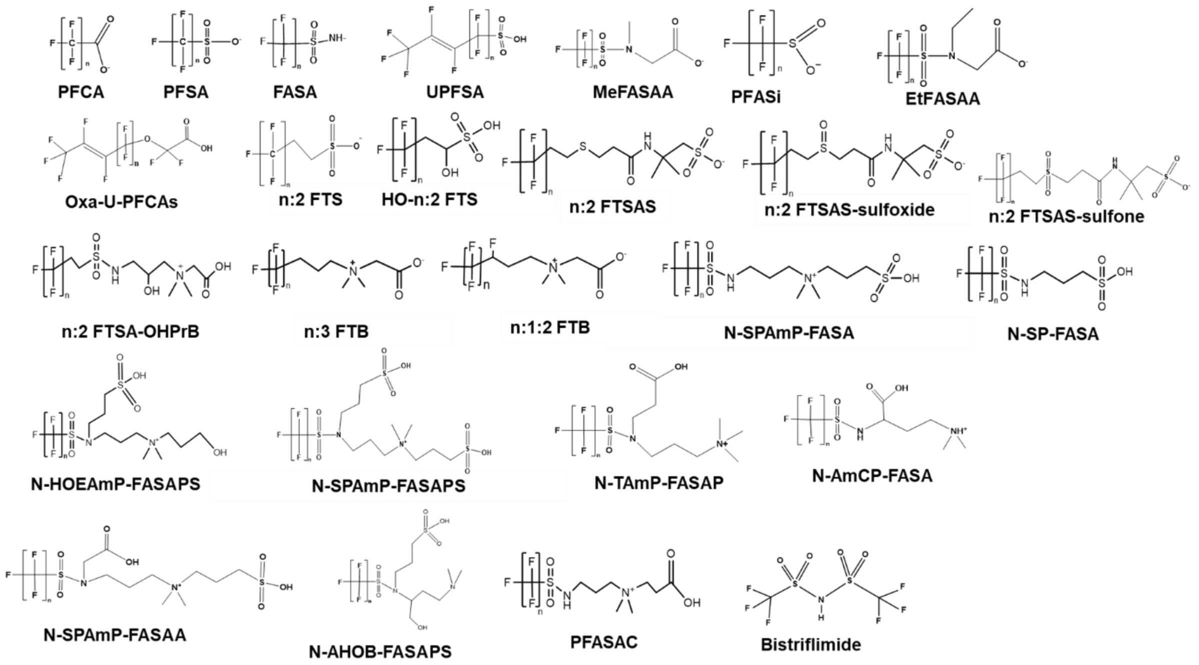

Figure S8. Proposed structures of bistriflimide and other key PFAS classes analyzed in this study.

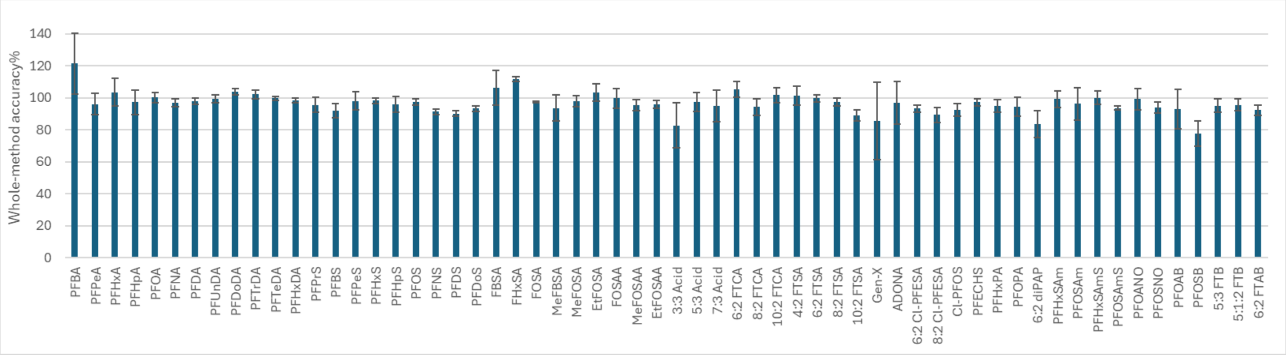

Figure S9. Accuracy of matrix spikes to surface water matrix, wherein native PFAS and surrogate internal standards were spiked to a Canadian river water sample before automated SPE and LC-HRMS analysis [9].

Note: We recognize that because of the distance between the sampling sites (in Lyon, France) and analytical facilities (at Université de Montréal, Canada), and considering the sample size required (500 mL per sample), it was not feasible to obtain additional surface water samples of France devoted to calibration curve construction. However, based on the accuracy of matrix spikes to Canadian surface water, the use of an extracted calibration curve (submitted to SPE), and the fact that surrogate internal standards also imparted correction, we are confident that the approach used here to quantify French water samples still provided valid results.

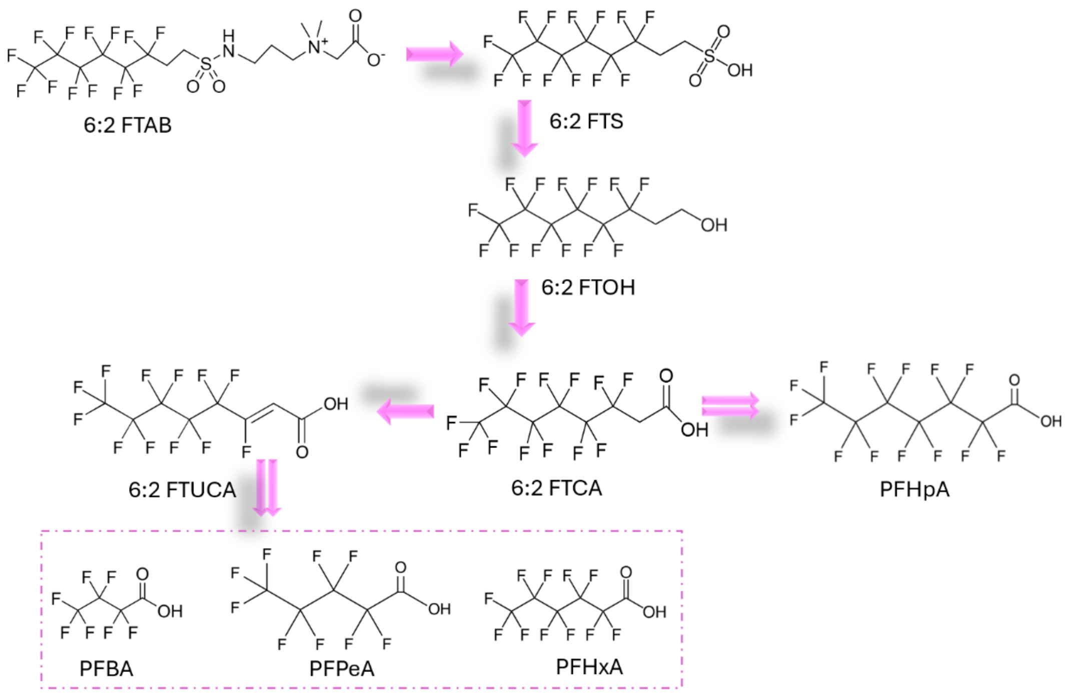

Figure S10. Potential degradation transformation pathway of 6:2 FTAB into 6:2 FTS followed by further degradation into Short-Chain PFCAs [10, 11].

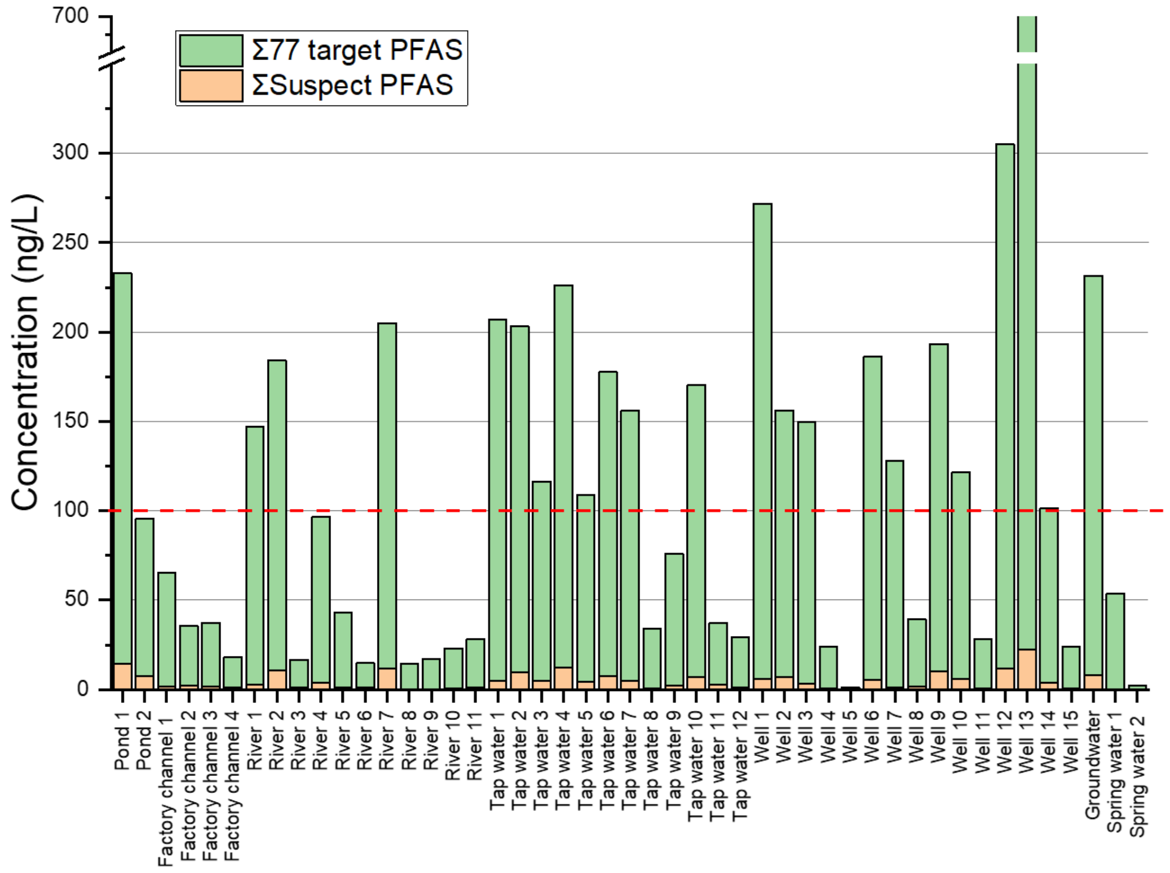

Figure S11. Concentrations of Σ_{77} target PFAS (green bars) and Σ_{121} suspect PFAS (orange bars) across various water sources in southern Lyon. The red dashed line indicates the 100 ng/L threshold by the European Union, although this European Union limit applies specifically to 20 PFAS in drinking water.

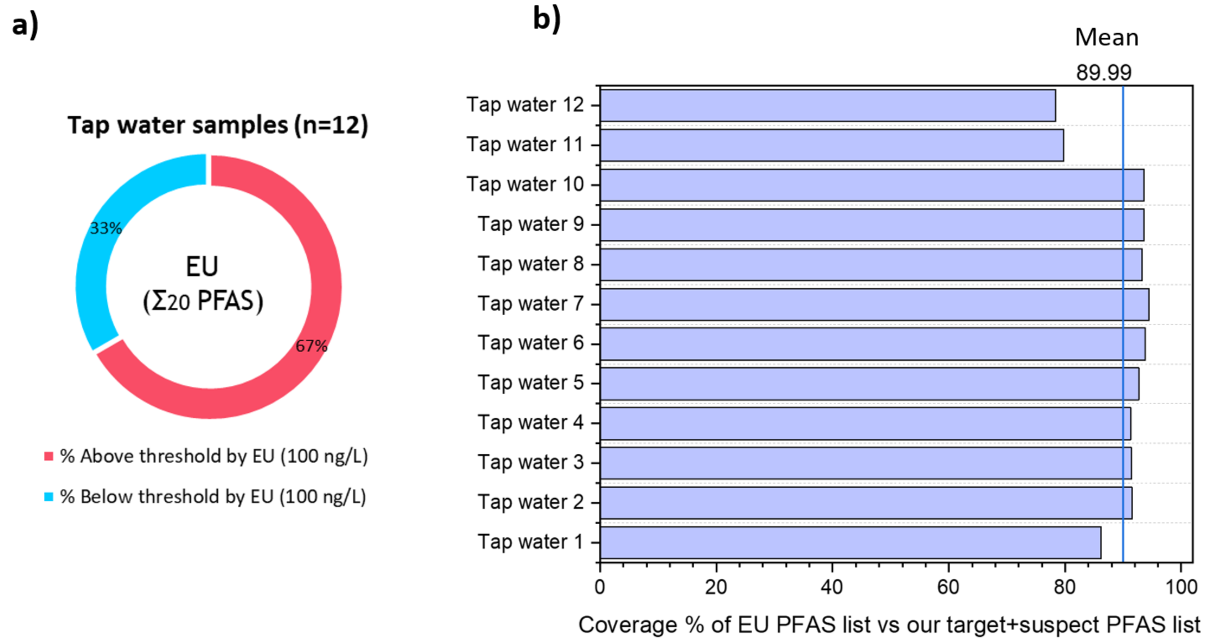

Figure S12. a) The doughnut chart shows the percentage of tap water samples with Σ_{20}PFAS concentrations above EU threshold (100 ng/L for Σ_{20}PFAS); **b)** The bar charts illustrate the coverage percentages of the Σ_{20}PFAS from EU guideline for drinking water in comparison to our Σ_{198} target+suspect screening PFAS among the analyzed tap water samples in this study.

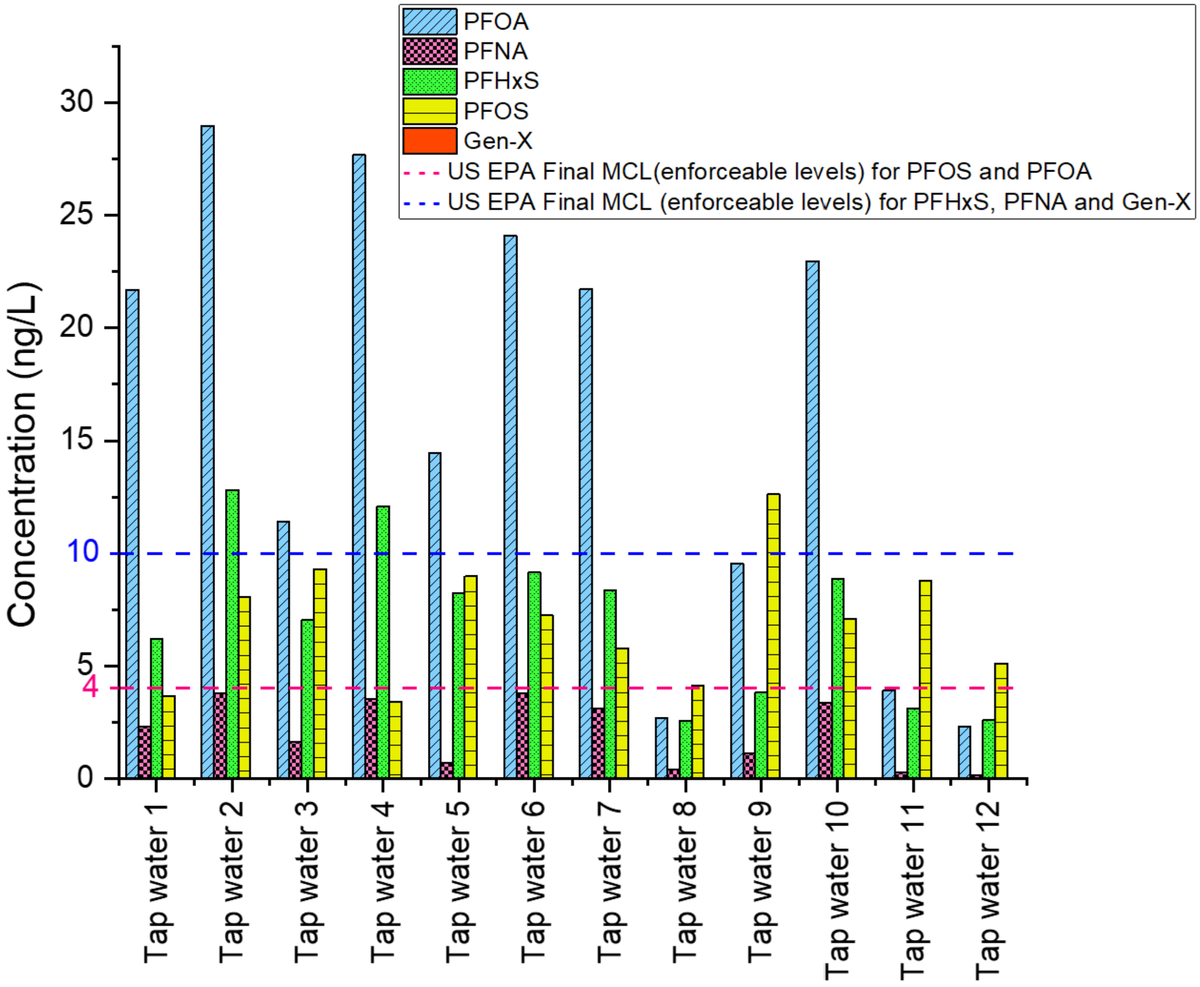

Figure S13. Concentrations of five PFAS compounds (PFOA, PFNA, PFHxS, PFOS, and Gen-X) in tap water samples. The red dashed line represents the US-EPA final maximum contaminant enforceable level (MCL) of 4 ng/L for PFOS and PFOA, while the blue dashed line indicates the US-EPA final MCL of 10 ng/L for PFHxS, PFNA, and Gen-X.

References

1. Liu, M., et al., *Hunting the missing fluorine in aqueous film-forming foams containing per-and polyfluoroalkyl substances*. Journal of Hazardous Materials, 2024. **464**: p. 133006.
2. Schymanski, E.L., et al., *Identifying small molecules via high resolution mass spectrometry: communicating confidence*. 2014, ACS Publications.
3. Kaboré, H.A., et al., *Worldwide drinking water occurrence and levels of newly-identified perfluoroalkyl and polyfluoroalkyl substances*. Science of The Total Environment, 2018. **616**: p. 1089-1100.
4. Nickerson, A., et al., *Spatial trends of anionic, zwitterionic, and cationic PFASs at an AFFF-impacted site*. Environmental Science & Technology, 2020. **55**(1): p. 313-323.
5. Liu, M., et al., *Per-and polyfluoroalkyl substances in contaminated soil and groundwater at airports: a Canadian case study*. Environmental Science & Technology, 2021. **56**(2): p. 885-895.
6. Munoz, G., et al., *Analysis of zwitterionic, cationic, and anionic poly-and perfluoroalkyl surfactants in sediments by liquid chromatography polarity-switching electrospray ionization coupled to high resolution mass spectrometry*. Talanta, 2016. **152**: p. 447-456.
7. Barola, C., et al., *Untargeted Screening of Per-and Polyfluoroalkyl Substances (PFASs) in Airborne Particulate of Three Italian E-Waste Recycling Facilities*. Separations, 2023. **10**(11): p. 547.
8. Chow, S.J., et al., *Comparative investigation of PFAS adsorption onto activated carbon and anion exchange resins during long-term operation of a pilot treatment plant*. Water Research, 2022. **226**: p. 119198.
9. Munoz, G., et al., *Bioaccumulation and trophic magnification of emerging and legacy per-and polyfluoroalkyl substances (PFAS) in a St. Lawrence River food web*. Environmental Pollution, 2022. **309**: p. 119739.
10. Fang, B., et al., *Fluorotelomer betaines and sulfonic acid in aerobic wetland soil: Stability, biotransformation, and bacterial community response*. Journal of Hazardous Materials, 2024. **477**: p. 135261.
11. LaFond, J.A., et al., *Bacterial transformation of per-and poly-fluoroalkyl substances: a review for the field of bioremediation*. Environmental Science: Advances, 2023. **2**(8): p. 1019-1041.
